# Stable isotope analysis of human bone from Ganj Dareh, Iran, ca. 10,100 calBP

**DOI:** 10.1371/journal.pone.0247569

**Published:** 2021-03-02

**Authors:** Deborah C. Merrett, Christina Cheung, Christopher Meiklejohn, Michael P. Richards

**Affiliations:** 1 Department of Archaeology, Simon Fraser University, Burnaby, BC, Canada; 2 EA–Eco-anthropologie (UMR 7206), Muséum National d’Histoire Naturelle, CNRS, Université Paris Diderot, Paris, France; 3 UMR 7269, LAMPEA, Aix-Marseille Université, CNRS, Minist Culture, Aix-en-Provence, France; 4 Department of Anthropology, University of Winnipeg, Winnipeg, MB, Canada; Museo delle Civiltà, ITALY

## Abstract

We report here on stable carbon, nitrogen, and sulfur isotope values from bone collagen of human (n = 20) and faunal (n = 11) remains from the Early Neolithic site of Ganj Dareh, Iran, dating to ca. 10,100 cal. BP. Our focus explores how isotope values of human bone vary by age and sex, and evaluates dietary practices at this site. It also provides a baseline for future studies of subsistence in the early Holocene Central Zagros Mountains, from the site with the first evidence for human ovicaprid management in the Near East. Human remains include individuals of all age groups for dietary reconstruction, as well two Ottoman intrusive burials for temporal and cultural comparison. All analyzed individuals exhibited *δ*^13^C and *δ*^15^N values consistent with a diet based heavily on C_3_ terrestrial sources. There is no statistically significant difference between the isotopic compositions of the two sexes, though males appear to show larger variations compared to females. Interesting patterns in the isotopic compositions of the subadults suggested weaning children may be fed with supplements with distinctive *δ*^13^C values. Significant difference in sulfur isotope values between humans and fauna could be the earliest evidence of transhumance and could identify one older adult male as a possible transhumant shepherd. Both Ottoman individuals had distinctively different *δ*^13^C, *δ*^15^N, and *δ*^34^S values compared to the Neolithic individuals. This is the first large scale analysis of human stable isotopes from the eastern end of the early Holocene Fertile Crescent. It provides a baseline for future intersite exploration of stable isotopes and insight into the lifeways, health, and processes of neolithisation associated with the origins of goat domestication at Ganj Dareh and the surrounding Central Zagros.

## Introduction

Although the Levant and Anatolia have received substantial attention concerning the origins of agriculture, much less has been reported on the easternmost Fertile Crescent, the Central Zagros. The ‘accepted’ story for this region revolves around one of the key processes of neolithisation: domestication of ovicaprids, and especially goat, a transformation suggested to have been unwittingly orchestrated by altered interactions between humans and local ungulate populations [1–4].

While recent excavations at the slightly earlier site of Sheik-e Abad address origins of herding through identification of penning and dung [5, 6], previous research was formulated using ovicaprid age and sex profiles as a proxy for human-goat interactions [2, 4, 7]. The first evidence for human control of goats comes from Ganj Dareh, where Hesse saw a predominance of young male goats/sheep in the deposits, i.e. slaughter pattern analysis, as supporting the hypothesis that the animals were under human control, managed rather than hunted [4, 8, 9]. Goat hoof impressions in mud bricks [10] also suggested that, though morphologically wild, the Ganj Dareh goats lived in close proximity to the human settlement and, at least behaviourally, were on the way to domestication [4, 8, 9]. Recent radiocarbon dates confirm contemporaneity of the human population [11] and ovicaprids [4] at the site, emphasizing the need to further explore life in the Early Neolithic of the Central Zagros. This study provides a new avenue for elucidating human lifeways at the site, with the first stable isotope analysis of the Ganj Dareh human remains. Published data from nearby contemporaneous sites are also included to provide a more comprehensive picture of dietary practices in the region.

### Introduction to the site of Ganj Dareh

**Fig 1:**
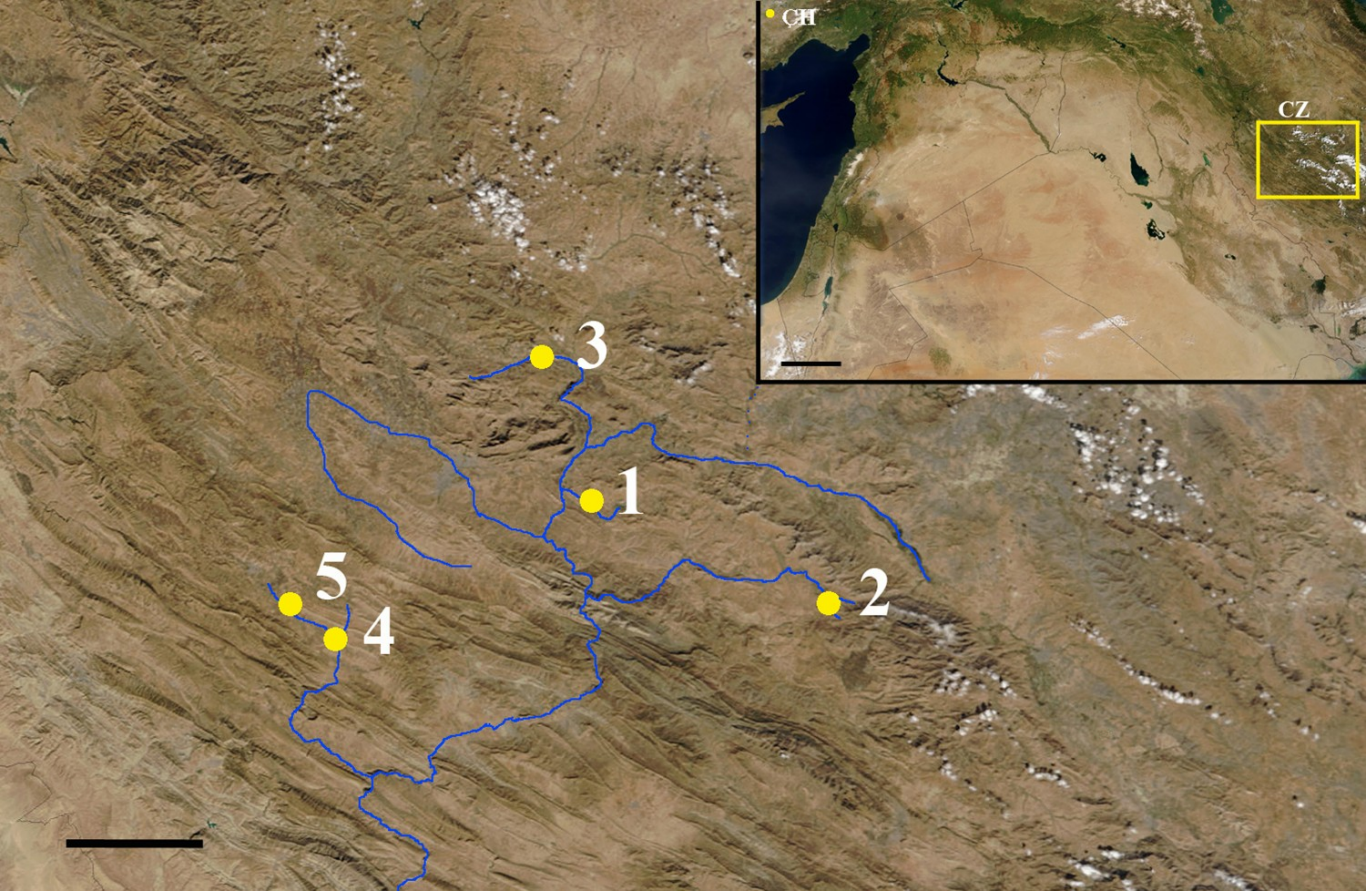
Location of Ganj Dareh (1) and other sites of Central Zagros mentioned in the text: Abdul Hosein (2), Sheikh-e-Abad (3), Jani (4), and Wezmeh Cave (5). Inset: Location of Central Zagros (CZ) and Çatalhöyük (ÇH). Scale = 30 km; inset scale = 150 km. Modified from https://www.visibleearth.nasa.gov/images/106461/the-middle-east.

Ganj Dareh Tepe, a small mound or Tepe in the High Zagros of Kermanshah Province in Western Iran, is one of several in the area (Fig 1). Lying in the Gamas-Ab Valley at an altitude of ~1400 m [12], it measures ~40 m in diameter with 7 to 8 m of cultural deposits. The initial work identified five levels, A to E, with level E at the base and the first permanent architecture in level D, well preserved by an extensive fire. New research based on re-examination of the original field notes of Philip Smith, shows the stratigraphy of the site to be more complex than previously published [13] but does not alter any of the conclusions of this paper. During excavations sponsored by the University of Toronto and the Royal Ontario Museum, Philip E.L. Smith excavated roughly 20 percent of the mound in four seasons between 1967 and 1974 [10, 14–16]. Current evidence places site occupation at 10,170–9,700 cal. BP, based on goat remains [4] and ca. 10,100 cal. BP based on human remains [11], confirming the relatively short occupation period of the site (100–200 years), and the contemporaneity of the humans and ovicaprids. Analysis of the full radiocarbon record also lays to rest the earlier idea of a major hiatus between levels D and E at the base of the site [4, 11].

Carbonized macrobotanical remains were isolated during excavation [17], with two-row hulled barley (*Hordeum spontaneum*), a C_3_ crop, the only cereal isolated. However, general poor preservation made it clear that presence of low levels of non-shattering rachis morphology alone was not sufficient to identify domestication [18]; at Early Neolithic sites of the Euphrates Valley with excellent plant preservation, non-shattering internode appearance was identical to that of modern wild populations. Thus, while there is insufficient evidence for domestication of barley, presence of barley and grinding stones suggests, at a minimum, the use of wild plants [19].

As with the botanical remains [17], faunal proportions did not vary through the occupation sequence, suggesting that subsistence did not change during use of the site [2, 4, 11]. Though a broad faunal spectrum was in use, with 49 identifiable taxa; goats, sheep, fox and partridge predominated and, of identifiable mammalian bone, 89.9% were ovicaprids, with goat predominating [7]. All levels displayed slaughter patterns consistent with goat management rather than hunting [2].

### Principals of stable isotope analysis

Stable carbon and nitrogen isotope analysis of bone collagen, expressed as *δ*^13^C and *δ*^15^N, is in routine use for dietary and mobility reconstruction [20–23]. More recently, analysis of the stable sulfur isotope (*δ*^34^S) has become a significant tool in reconstructing ancient lifeways [24]. The stable carbon isotope ratio (^13^C/^12^C) distinguishes organisms exploiting marine, terrestrial, and plant based ecosystems, with their different photosynthetic pathways (e.g. C_3_ vs. C_4_) [25–27]. The stable nitrogen isotope ratio (^15^N/^14^N) identifies trophic level effects in the food chain, with a 3–5‰ enrichment from one trophic level to another, making *δ*^15^N values particularly useful for estimating animal protein intake [28]. However, they are also impacted by physiological and environmental phenomena, such as breastfeeding [29], long-term starvation [30], manuring [31, 32], and aridity [33, 34]. The stable sulfur isotope ratio (^34^S/^32^S) is primarily influenced by geochemical conditions [35] and, while highly variable and locally specific, can be used to identify migrants from regions with distinctive *δ*^34^S baselines. The primary sources of sulfur in bone are dietary methionine, and its internal recycling. Methionine is an essential amino acid. Thus, *δ*^34^S values reflect methionine from dietary protein [24], and may also contribute to health assessment by providing insight into nitrogen metabolism and stress exposure.

## Materials

Samples were collected from 32 human and 25 faunal bones (Table 1). 30 human samples are Early Neolithic (ca. 10,100 cal. BP). An additional two (GD# 1150 and GD# 1151–1), surface burials of Ottoman age (450–320 years cal. BP) [11], are included for the record. The human remains were from burials beneath house floors, in the rubble that accumulated in collapsed houses and, in one case, from a test pit outside the mound.

**Table 1:** Human and faunal samples analysed showing level in the site and burial context relative to architectural features.

Ganj Dareh #	Species	Age-at-Death	Sex	Site Level	Burial Context
**#4**	Human	MA	F?	B	Pit in D burnt rubble
**#7**	Human	0–1 mo		A/B	Pit in B rubble
**#10**	Human	8.5–9.5 yr		A/B	Above soil of D
**#12**	Human	1 mo▲		D	Below floor
**#13a**	Human	MA	F*	C	On floor of mud brick structure
**#14b**	Human	2.5–4 yr	F*	B/C	Pit in burnt D rubble
**#15**	Human	15 yr		C	In small mud brick chamber
**#16**	Human	5.5–7.5 yr		C	In small mud brick chamber
**#20**	Human	YA	M	D	Under house floor
**#22**	Human	OA	M*	D	Under house floor
**#23**	Human	YA	F	C	Below mud brick-floored structure
**#25**	Human	7 mo▲	F*	D	Under house floor
**#31**	Human	MA	M	D	Outside settlement, mixed fill
**#34**	Human	OA	M?	D	Mixed fill, below level D floor
**#36**	Human	1.1 yr▲		D	In stone-lined chamber
**#37**	Human	MA	M	D	Below level D floor
**#38**	Human	2.5–3 yr		D	Beside level D ‘kiln’
**#40**	Human	YA	M*	D	Beside level D ‘kiln’
**#1150**	Human	YA	F*	surface	From faunal units
**#1151–1**	Human	MA		surface	From faunal units
**#12**	dog			D	#12
**#13**	fauna			C	#13
**#17**	fauna			C	#17
**#20**	fauna			D	#20
**#25**	fauna			D	#25
**#31**	fauna			D	#31
**#34**	fauna			D	#34
**#37**	fauna			D	#37
**#40**	fauna			D	#40
**#1150**	fauna			surface	#1150
**#1151–1**	fauna			surface	#1151–1

▲ = enamel cross-striation ages [36, 37].

* = genetic sex determination [38, 39]. M = male, F = female. YA = young adult, MA = middle adult, OA = older adult.

In general, the human bone collection is highly fragmentary, leading to differing MNI estimates over the course of the reconstruction and analysis of the collection. Factors included identification of multiple burials over several rounds of analysis and recovery of 17 isolated single human bones/bone fragments/single teeth from the faunal sample. The current MNI is 116, with 56 catalogued as skeletons, defined as those with >4 skeletal elements [36]. Of these, 52 were identified during the excavation process [40]. For biological and metabolic consistency of bone used and minimal bone destruction, ribs were only sampled from individuals classified as skeletons (N = 32 with ribs present). No permits were required for this study, which complied with all relevant regulations. Samples are curated by one of us (DCM) at Department of Archaeology, Simon Fraser University. The faunal remains used in this study were included in the work of Hesse (7–9), then associated in the collection with the human assemblage as fauna accompanying the human burials. Although probably not intentionally associated with the human remains, they none-the-less were within the same stratigraphic level and in close enough proximity to be identified as with the human remains.

The internal site structure gave no evidence for courtyards or roads; remains were recovered from within architectural features, primarily below house floors or in bricked features [10]. A large expanse of level D was exposed to fire, preserving mud brick walls up to a metre in height, with some below-house-floor burials sufficiently exposed to heat to cause bone calcination [10]. This reduced the number of successful collagen extractions in the present study.

## Methods

### Osteological analysis

Ages-at-death estimations for the human skeletal remains are by Merrett [36, 37]. Non-adult ages were based on multiple methods: tooth development and eruption [41, 42], enamel cross-striation age [43], bone developmental patterns [44], bone length [44–49], and patterns of epiphyseal fusion [44, 50, 51]. Adult age estimation methods included tooth wear [52, 53], pubic symphysis [54], auricular surface morphology [55], and cranial and palatal suture closure [56, 57]. Adult sex estimates were based on sexual dimorphism of cranial and pelvic bones [50, 58–60]. Sex of six individuals was determined through aDNA analysis [38, 39]. Bone samples for stable isotope analysis were from rib fragments. In addition, although most human remains were from below-house floor contexts, two were identified as from midden-like sediments: one within roof rubble, the other outside the settlement (Table 1).

Faunal remains used here were excavated as ‘associated’ with the human remains, and part of the sample analysed and placed in the unidentified category by Hesse [7]. The original excavation notes give no evidence for ritual interment of fauna. Although the fauna were too fragmented for identification, all used were from the ’medium mammal long bone’ category, and were most likely ovicaprid. As a result, the faunal isotopic results can only serve as a regional baseline, and cannot be used to evaluate the dietary compositions of the Ganj Dareh residents.

### Stable isotope analysis

Bone samples were prepared at the Isotope Chemistry Laboratory, Simon Fraser University; extracted collagen was sent to Isoanalytical Limited (UK) for analysis. Collagen was extracted following the modified Longin method [61] and additional ultrafiltration. Sample analysis was with a Europa Scientific^TM^ elemental analyzer, coupled to a mass spectrometer. All carbon and nitrogen isotope values are averaged, based on duplicate analysis, and reported in per mil (‰). Results are calibrated to VPDB and AIR, respectively, using international standards IAEA-N-1, IAEA-C7, and IAEA-CH-6. Accuracy of measurements are monitored using in-house check standards IA-R068 (soy protein, *δ*^13^C = –25.22‰, *δ*^15^N = +0.99‰), IA-R038 (L-alanine, *δ*^13^C = –24.99‰, *δ*^15^N = –0.65‰), IA-R069 (tuna protein, *δ*^13^C = –18.88‰, *δ*^15^N = +11.60‰), and a mixture of IA-R046 and IAEA-C7 (ammonium sulfate and oxalic acid, *δ*^13^C = –14.48‰, *δ*^15^N = +22.04‰). Averaged measured *δ*^13^C values for IA-R068 (n = 12), IA-R038 (n = 5), IA-R069 (n = 5), and IA-R046/IAEA-C7 (n = 5) are –25.23‰± 0.04‰, –25.04‰± 0.03‰, –18.90‰± 0.02‰, and –14.53‰± 0.06‰, respectively. Averaged measured *δ*^15^N values for IA-R068 (n = 12), IA-R038 (n = 5), IA-R069 (n = 5), and IA-R046/IAEA-C7 (n = 5) are +1.00‰± 0.04‰, –0.59‰± 0.04‰, +11.71‰± 0.04‰, and +21.89‰± 0.09‰, respectively.

For sulfur isotope analysis, all measurements are reported in per mil (‰). Results are calibrated to VCDT using international standards NBS-127, IAEA-S-1, and IAEA-SO-5. Accuracy of measurements are monitored using in-house check standards IA-R061 (barium sulfate, *δ*^34^S = +20.33‰), IA-R069 (tuna protein, *δ*^34^S = +18.91‰), and NBS-1577B (bovine liver, *δ*^34^S = +7.50‰). Averaged measured *δ*^34^S values for IA-R061 (n = 6), IA-R069 (n = 3), and NBS-1577B are +20.31‰±0.08‰, +18.69‰±0.31‰, and +7.61‰±0.20‰, respectively.

Collagen quality was assessed using conventional criteria: %collagen between 0.5% and 22% by weight, %C between 15.3% and 47%, %N between 5.5% and 17.3%, %S between 13% and 35%, atomic C/N ratio between 2.9 and 3.6, atomic C/S ratio between 300 and 900, and atomic N/S ratio between 100 and 300 [61–67]. Only samples with elemental compositions within these ranges were accepted for analysis.

## Results

With many samples heavily calcined, only 20 of 32 human and 11 of 25 faunal samples yielded sufficient preserved collagen for C and N measurements; 10 of 20 human and 10 of 11 faunal samples for S measurements. Results of accepted measurements, elemental compositions, and sample information are listed in Table 2.

**Table 2:** Summary of sample information, elemental compositions, and bone collagen carbon, nitrogen, and sulfur isotopic compositions of all samples analysed in this study.

S_SFU #	Site ID #	Collagen (%)	Species	Age	Sex	C (%)	N (%)	S (%)	C/N	C/S	N/S	*δ*^13^C (‰)	*δ* ^15^N (‰)	*δ* ^34^S (‰)
**123**	**#4**	3.53%	Human	MA	F?	42.40	14.32	0.20	3.5	571.1	165.4	–19.6	+10.3	+11.0
**124**	**#7**	7.97%	Human	0–1 mo		42.21	14.27	0.21	3.5	536.1	155.4	–19.6	+11.4	+9.9
**125**	**#10**	1.13%	Human	8.5–9.5 yr		18.45	5.90		3.6			–20.4	+10.5	
**126**	**#12**	4.44%	Human	1 mo		25.83	8.50		3.5			–19.6	+11.7	
**127**	**#13a**	5.09%	Human	MA	F	29.04	9.99	0.15	3.4	512.1	151.0	–19.7	+10.2	+10.5
**128**	**#14b**	9.21%	Human	2.5–4 yr	F*	41.87	14.36	0.17	3.4	644.8	189.7	–19.2	+11.0	+10.4
**129**	**#15**	2.08%	Human	15 yr		11.52	3.80		3.5			–20.1	+10.3	
**130**	**#16**	6.07%	Human	5.5–7.5 yr	M*	40.62	13.91	0.17	3.4	621.2	182.4	–19.5	+10.0	+9.7
**132**	**#20**	5.91%	Human	YA	M	38.64	13.19	0.22	3.4	464.1	135.9	–20.0	+10.8	+11.0
**134**	**#22**	5.62%	Human	OA	M	42.69	14.83		3.4			–19.6	+10.3	
**135**	**#23**	5.43%	Human	YA	F	43.21	14.97	0.24	3.4	478.9	142.2	–19.8	+10.5	+11.6
**136**	**#25**	4.51%	Human	7 mo	F*	39.81	13.53		3.4			–18.3	+14.3	
**141**	**#31**	1.29%	Human	MA	M	22.19	7.18		3.6			–20.1	+11.4	
**144**	**#34**	5.49%	Human	OA	M?	39.13	13.49	0.25	3.4	416.9	123.2	–19.2	+9.6	+14.2
**145**	**#36**	8.17%	Human	1.1 yr		41.53	14.60	0.26	3.3	421.0	126.9	–19.2	+12.9	+12.9
**146**	**#37**	5.83%	Human	MA	M	27.91	9.68		3.4			–19.4	+10.0	
**147**	**#38**	3.35%	Human	2.5–3 yr		24.08	8.18		3.4			–19.6	+14.4	
**149**	**#40**	5.67%	Human	YA	M	33.20	11.66		3.3			–19.9	+10.1	
**151**	**#1150**	3.30%	Human	YA	F	37.01	12.74	0.22	3.4	441.0	130.1	–18.2	+8.7	+9.0
**152**	**#1151–1**	2.82%	Human	MA		30.48	10.44		3.4			–18.3	+9.9	
**157**	**#12**	2.73%	Dog			38.84	13.13	0.28	3.4	371.7	107.7	–20.8	+9.9	+12.4
**158**	**#13**	6.16%	Unident. fauna			36.80	13.16	0.16	3.3	610.5	187.2	–20.1	+8.7	+13.8
**161**	**#17**	5.48%	Unident. fauna			34.84	12.83	0.27	3.2	350.7	110.7	–19.0	+8.4	+14.1
**162**	**#20**	4.29%	Unident. fauna			32.14	11.67		3.2			–20.0	+7.1	
**166**	**#25**	5.89%	Unident. fauna			38.01	13.13	0.25	3.4	406.9	120.5	–20.1	+7.4	+13.1
**170**	**#31**	4.46%	Unident. fauna			39.74	14.39	0.19	3.2	546.6	169.7	–18.8	+10.3	+11.5
**172**	**#34**	9.44%	Unident. fauna			42.91	15.04	0.20	3.3	569.4	171.1	–19.0	+9.7	+14.0
**173**	**#37**	5.10%	Unident. fauna			36.02	12.34	0.18	3.4	542.2	159.3	–20.2	+6.9	+14.2
**174**	**#40**	4.09%	Unident. fauna			39.88	13.65	0.19	3.4	546.7	160.4	–19.7	+7.1	+13.1
**176**	**#1150**	4.41%	Unident. fauna			28.02	9.53	0.19	3.4	401.7	117.2	–19.7	+7.1	+12.8
**177**	**#1151**	4.32%	Unident. fauna			40.33	13.98	0.18	3.4	611.2	181.7	–19.7	+8.2	+13.1

Age: YA = 18-29yr, MA = 30–50 yr, OA = >50 years. Sex: Skeletal sex for adults: F = female, M = male, F? = possible F, M? = possible M.

* = sex given for nonadults was genetically determined [38, 39].

**Fig 2:**
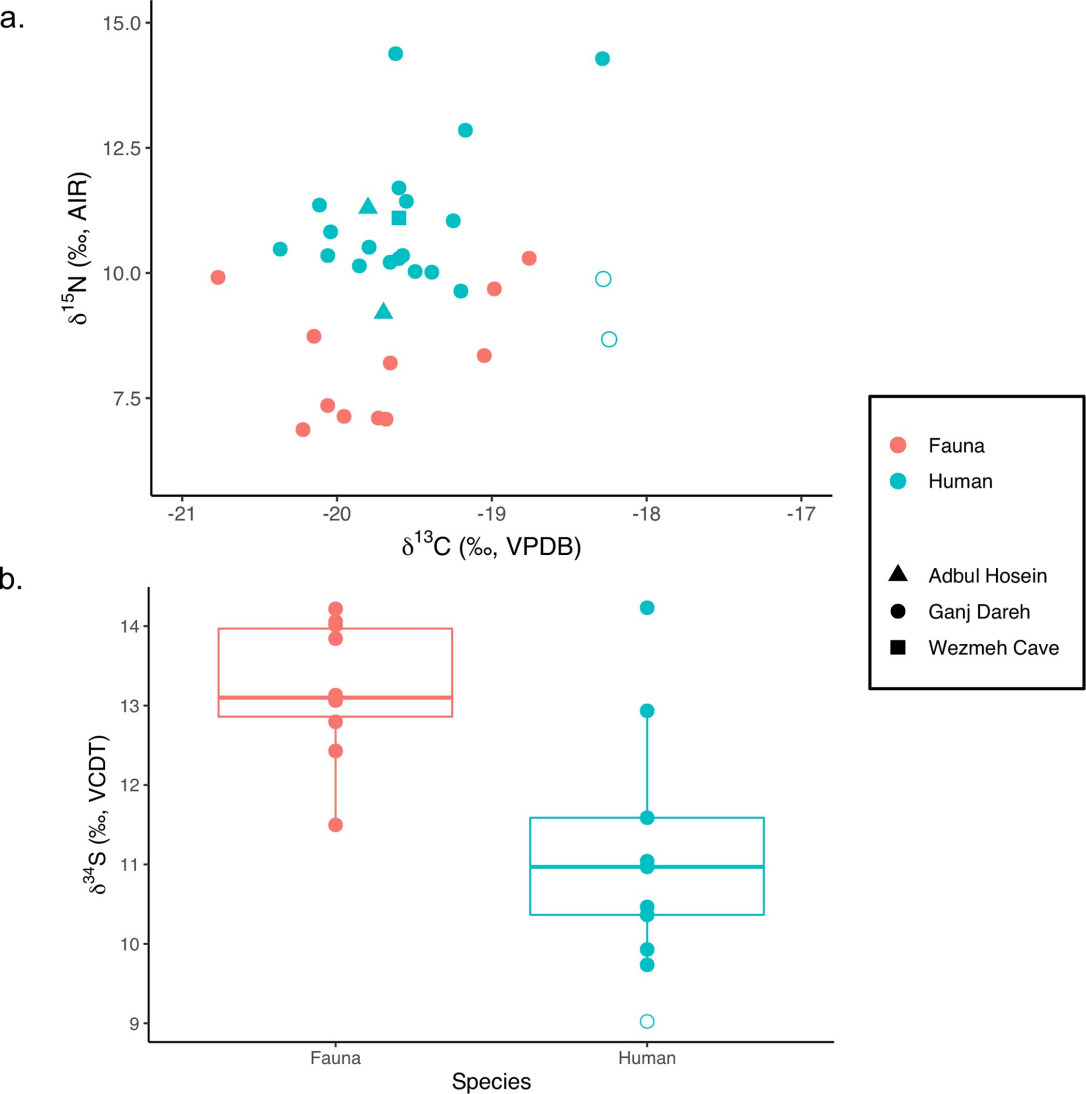
Human and faunal stable carbon, nitrogen, and sulfur isotope data from western Iran. Closed circles (●) correspond to Neolithic samples, open circles (○) correspond to human samples dated to the Ottoman period: a) carbon and nitrogen isotope data of all humans and fauna from western Iran; data from Adbul Hosein and Wezmeh Cave are from [68]; b) sulfur isotope data of all humans and fauna from Ganj Dareh.

Human and faunal C, N, and S measurements are plotted in Fig 2, together with published human C and N data (n = 3) from the nearby and contemporary sites Abdul Hosein (AH) and Wezmeh Cave (WC) (2 and 5 in Fig 1). All humans have *δ*^13^C values between –20.4 and –18.2‰, *δ*^15^N between +8.7 and +14.4‰, and *δ*^34^S between +9.0 and +14.2‰. All fauna have *δ*^13^C values between –20.8 and –17.9‰, *δ*^15^N between +6.9 and +10.3‰, and *δ*^34^S between +11.5 and +14.2‰. *δ*^13^C and *δ*^15^N values of the three individuals from AH and WC fall well within the Ganj Dareh range. However, the two Ottoman intrusive burials (Fig 2) have distinctive stable isotopic compositions (C, N, and S) compared to the Ganj Dareh Neolithic group. Unfortunately, the Ottoman sample size is too small for meaningful discussion. S measurements are only available for Ganj Dareh (Fig 2B). The S compositions of the humans are significantly depleted in ^34^S compared to those of the fauna (Wilcoxon test: diff: 2.0‰, *p* = 0.01).

### Sex comparison

**Fig 3:**
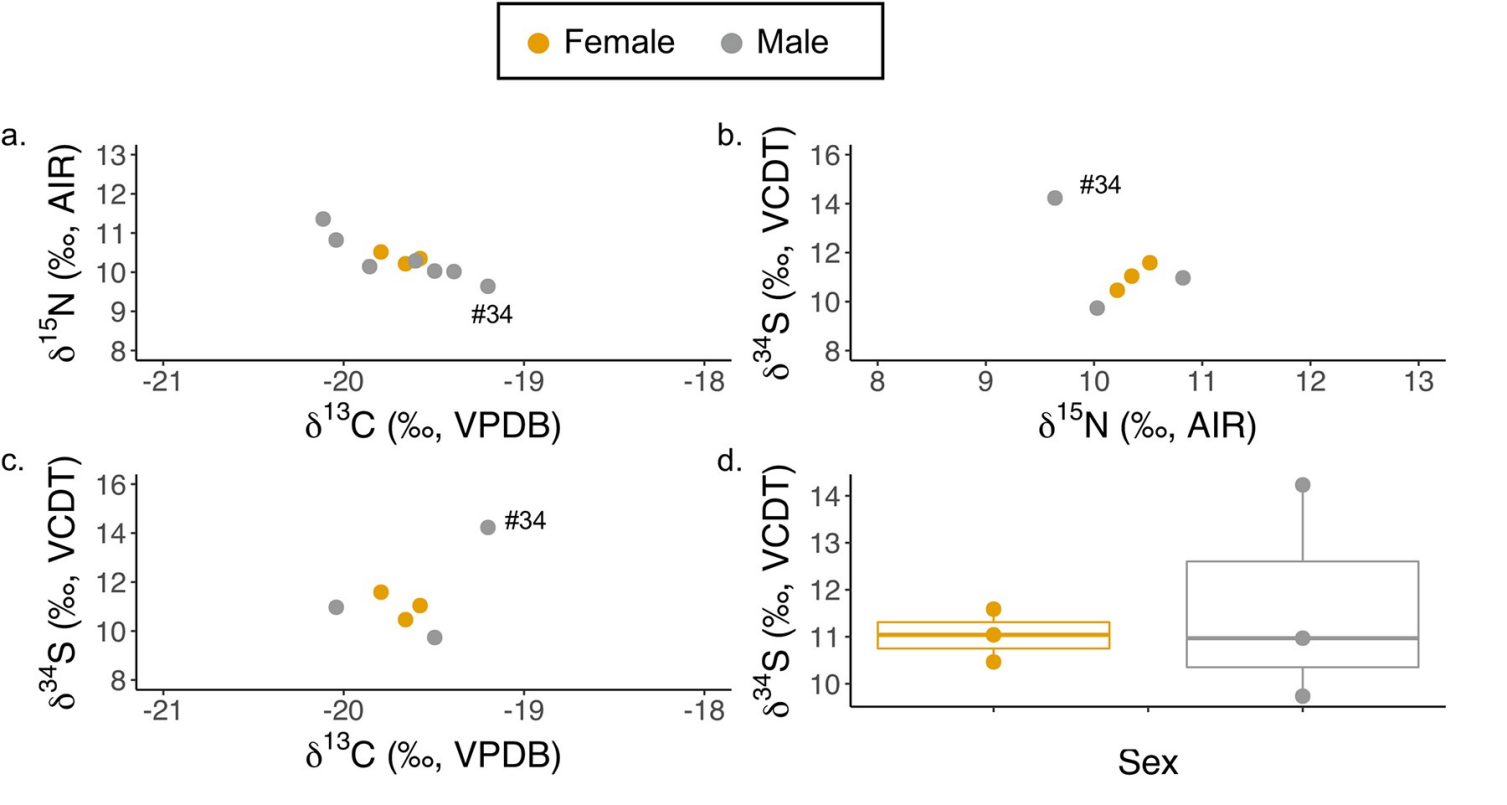
Stable carbon, nitrogen, and sulfur isotope data of all sexed individuals from Ganj Dareh: a) carbon and nitrogen isotope data; b) nitrogen and sulfur isotope data; c) carbon and sulfur isotope data; and d) the sulfur isotope data.

For this comparison, all “possible male” and “possible female” individuals are treated as “male” and “female”, respectively. Only Neolithic individuals above the age of 4 are considered, so that *δ*^15^N values will not be complicated by breastfeeding effect. *δ*^13^C, *δ*^15^N, and δ^34^S values do not significantly differ between males and females (Wilcoxon tests: *δ*^13^C: *W* = 10, *p* = 1.0; *δ*^15^N: *W* = 13, *p* = 0.5167; and δ^34^S: *W* = 5, *p* = 1.0) (Fig 3).

### Non-adult comparison

**Fig 4:**
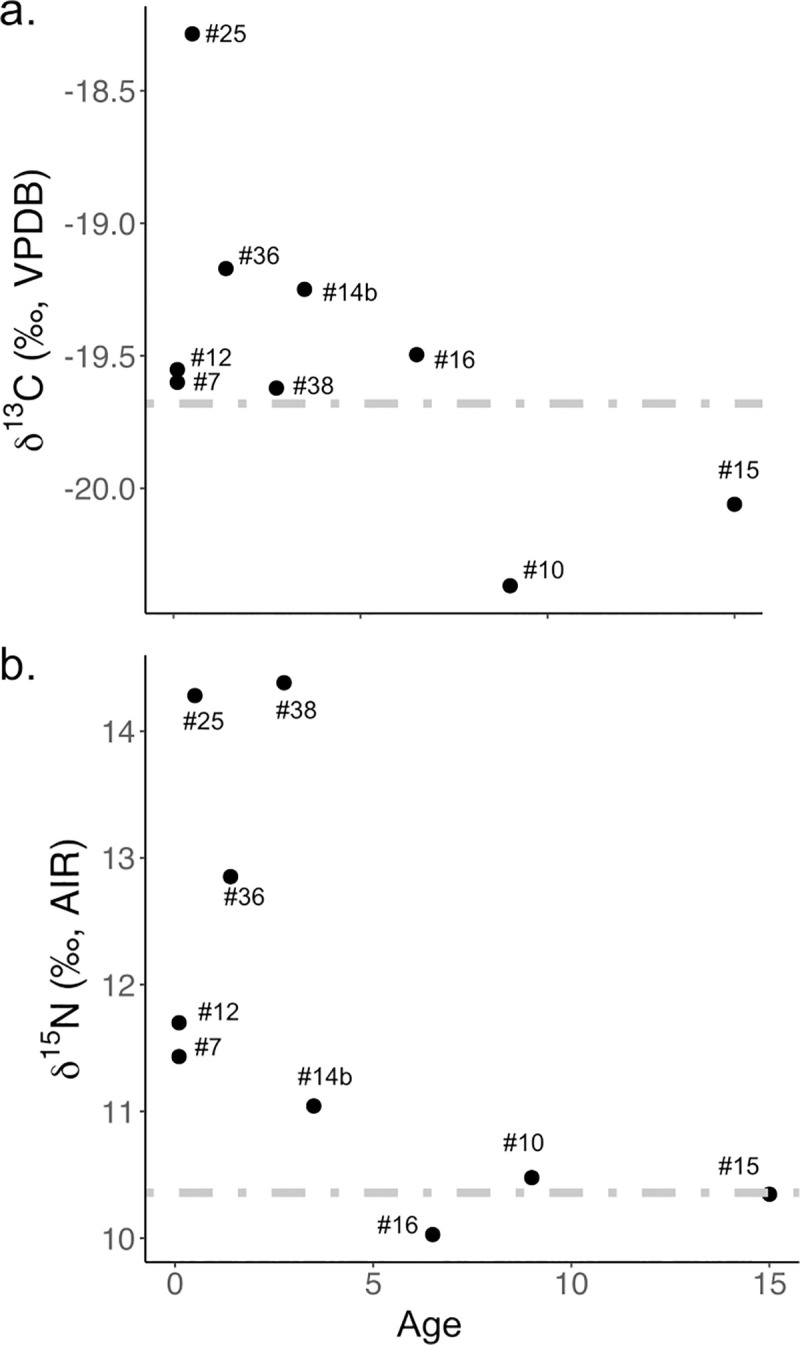
Carbon and nitrogen isotope data for Ganj Dareh non-adults (age < 15 years): a) carbon isotope values of non-adults relative to mean Neolithic adult female value (–19.7‰); b) nitrogen isotope values of non-adults relative to mean Neolithic adult female value (+10.4‰). Dotted lines correspond to mean female adult values.

Fig 4 shows the age profiles of stable carbon and nitrogen isotope values in non-adults (n = 9). Because we have no genetic evidence for mother/child relationships in the sample, the means of all adult females from the site (*δ*^13^C: –19.7‰; *δ*^15^N: +10.4‰, n = 3) are used as the baseline to evaluate the effect of breastfeeding in non-adults. Both *δ*^13^C and *δ*^15^N values decrease with increasing age.

## Discussion

The overall *δ*^13^C and *δ*^15^N human and fauna values from Ganj Dareh provide an initial baseline for the Early Neolithic of the Central Zagros and are consistent with the limited data available from nearby Adbul Hosein and Wezmeh Cave (Fig 2A). However, further research is needed before evaluation of the meanings of intersite variation can proceed. We need not expect the trajectory away from hunting and gathering to be linear, occur at similar rates, nor vary in similar ways between sites or even within one region [69]. These data are however invaluable in the exploration of the pathways to goat domestication in the Central Zagros, especially given that recent genetic findings [70] indicate the relative isolation of the region from processes of neolithisation elsewhere in the Fertile Crescent to the west.

### Sex comparison

While no statistically significant difference exists between the means of the two sexes, the males have much larger ranges for all three isotopes than do the females (Fig 3 and Table 3). Given the small sample size, any interpretation of higher male variability is speculative without further evidence. Some possible explanations include: i) males had access to a larger variety of foods, ii) males had more access to ‘exotic’ foods for ceremonial purposes, and iii) males had higher mobility than females. More than one of these processes may be occurring.

**Table 3:** Comparing variability in Ganj Dareh isotopic compositions between males and females (age 4+) using total range and standard deviation.

	Range (‰)		Standard deviation (‰)	
	Male	Female	Male	Female
***δ***^**13**^**C**	1.2	0.2	0.4	0.1
***δ***^**15**^**N**	1.7	0.3	0.5	0.2
***δ***^**34**^**S**	4.5	1.1	2.3	0.6

### Stable carbon and nitrogen isotope analysis

The Ganj Dareh fauna (only one specimen identified to species, dog) have *δ*^13^C (–20.8‰ to –18.8‰) and *δ*^15^N values (+6.9‰ to +10.3‰) consistent with those from contemporary Wezmeh Cave (5 in Fig 1) [66], Jani (4 in Fig 1) and Sheikh-e-Abad (3 in Fig 1) [71]. This contrasts with contemporary Çatalhöyük, at a much lower elevation on the Turkish Konya Plain and with a less continental climate. In this case, maximum faunal *δ*^13^C values are substantially higher than the Zagros values, at –13.6‰ for sheep and –12.5‰ for goats. Pearson and colleagues [72] suggest presence of C_4_ plants such as wet/salt tolerant grasses in the Çatalhöyük diet. The Ganj Dareh data suggest that either C_4_ grasses were not present or were not utilized in the early Holocene. Following the *δ*^13^C trend, minimum *δ*^15^N values from Çatalhöyük fauna are similar to those from Zagros sites while the maximum value for sheep is substantially higher (+14.4‰) [72], again illustrating different resource availability or utilization in the two regions.

Turning to the human material, adult *δ*^13^C and *δ*^15^N values from Neolithic Ganj Dareh are also in keeping with the one individual from Wezmeh Cave and the two from Abdul Hosein (Fig 2A), suggesting similar resource access and utilization in the Central Zagros. All individuals have a *δ*^13^C value range between –20.4‰ and –19.2‰ (n = 17), values consistent with a terrestrial diet based primarily on C_3_ resources, as noted above.

In archaeology, weaning practices in past populations can be detected using stable carbon and nitrogen isotope analysis. Previous studies have assumed that the enrichment in nitrogen isotope ratios from mother to infant is between 2–5‰ [73–77]. A smaller enrichment between 0.5 and 1.4‰ is also expected in the *δ*^13^C values of breastfeeding individuals [29]. In this study, similar pattern in both *δ*^13^C and *δ*^15^N values of the subadults can be seen (Fig 4), where the highest *δ*^13^C and *δ*^15^N values are reported amongst children below the age of 4. Unfortunately, our sample size is too small to allow for a more specific estimation of weaning age at the site.

There has been growing evidence that other than diet, high levels of stress exposure and malnutrition may also cause variability in the isotopic compositions of humans [29, 78–80], mainly in *δ*^15^N values [79]. However, at Ganj Dareh, the corresponding patterns in both *δ*^13^C and *δ*^15^N values of the subadults suggested that diet was a stronger factor than metabolic disruption for the trend in these individuals’ isotopic compositions.

### Stable sulfur isotope analysis

The difference in *δ*^34^S values between the Ganj Dareh human and faunal samples is quite unusual (Fig 2B). As mentioned earlier, *δ*^34^S values vary in different geological environments; *δ*^34^S values should reflect where food was grown and/or produced. These data suggest that, at Ganj Dareh, humans and fauna were exploiting different geographical regions. This could involve a specific animal husbandry regime, with animals raised in a different area, with a distinctive S isotope baseline compared to where plant foods were grown and/or collected. Alternative explanations include fauna being brought in from elsewhere for herd replacement following local faunal extinctions [36] and/or for funerary feasting events. A final possibility is that none of the faunal remains tested are from individuals or species that were part of the human diet with only one sample identified to species—dog, though the size profile of the faunal remains used and the frequency of various species demonstrated by Hesse [7–9] appears to make that unlikely.

Overall although sample size is small, similar to the patterns in *δ*^13^C and *δ*^15^N values, the male variation in *δ*^34^S values is much greater than that in females, suggesting possible sex differences. These could have origins in different food sources for men and women, both in regular diet and in ritual practices, matrilocal postmarital residence practices, or more variation in male adult travel. Consistent with the latter scenario, one adult male GD#34 has a very similar *δ*^34^S value to those of the fauna; he may have been closely associated with the animals. In the High Zagros, people practicing transhumance with goats could potentially spend much of the year in geologically different locations. Were this the case, then *δ*^34^S of GD#34 may be evidence during the early Neolithic for an early form of what later would become what is referred to as transhumance.

## Conclusions

We present the first large scale stable isotope analysis on human bone collagen from Ganj Dareh, Central Zagros, Iran, ca. 10,100 cal. BP. Though limited results have been previously published for other sites in the region, sample sizes were much smaller. As a result, our data establish a much more solid data set for future inter-site comparisons of lifeways for the Early Neolithic period of this region, and to test the hypothesis of multi-linear trajectories in the transition to agricultural subsistence enunciated by Smith’s scenario of ’low-level’ food production economies [69]. The carbon isotope measurements indicate a terrestrial C_3_-based diet, with no evidence of C_4_ plant usage, in strong support for the conclusion of van Zeist et al. [17].

Although not statistically different for all isotopes analysed, increased variation of males over females points to possible difference in dietary breadth between the sexes. Two children, aged 7 months and 2.5–3 years, have elevated *δ*^15^N values. This could indicate that they had not been weaned, but other explanations are possible, including exposure to stress episodes, or use of "special" weaning supplements.

Finally, sulfur isotope analysis identified an individual with an outlier *δ*^34^S value, an older adult male. We see the adult as either spending a considerable portion of his later life in a geologically non-local region, or at least ingesting non-local foods, consistent with possible interpretation of this individual as a transhumant shepherd. If so, the origins of pastoral transhumance may be earlier than previously thought.

Within the regional context of early neolithisation and incipient goat domestication, only three individuals from two other sites were available for comparison. Carbon and nitrogen measurements from these are consistent with those from Ganj Dareh. Given the considerably larger sample size for the Ganj Dareh analysis, we see the combined results as suggesting similar subsistence and lifeways across the Early Neolithic Central Zagros. Further investigations are indeed needed. It remains possible that life in the Central Zagros was one of ’low-level’ food production, wherein the trajectory from hunting/gathering to herding and farming was neither uniform within the region nor unidirectional [69]. What is also clearly needed is a sense of how our understanding of the archaeological models for the Early Neolithic, within a broader perspective, are affected by the recent aDNA evidence suggesting that the Ganj Dareh population had little if any biological relationship with contemporary and later groups in the Tigris/Euphrates lowlands or further west in the Levant, but were tied to groups to the north and northeast such as in the Caucasus (see especially [70]).
